# Asynchronous Online Platforms Can Reduce Healthcare Costs for STI and HIV Testing: Evaluating GetaKit.ca in Ontario, Canada

**DOI:** 10.1177/00469580261489227

**Published:** 2026-09-15

**Authors:** Patrick O’Byrne, Lauren Orser

**Affiliations:** 1 6363 University of Ottawa, School of Nursing, Ottawa, ON, Canada; 2 Ottawa Public Health, Sexual Health Clinic, Ottawa, ON, Canada

**Keywords:** testing, sexually transmitted infections, HIV, online systems

## Abstract

**Introduction:**

Rates of sexually transmitted infections (STIs) are increasing in Canada, while clinic capacity for testing and treatment remains constrained.

**Methods:**

To improve access to STI screening, we developed GetaKit.ca, a web-based platform in Ontario, Canada that allows individuals to complete an online risk assessment, receive STI testing recommendations, and access laboratory-based testing with follow-up through partnerships with local public health units. As part of the service evaluation, we conducted a two-year cost analysis comparing GetaKit with (1) public health STI clinic models in Ontario and (2) the publicly funded Ontario Health Insurance Plan (OHIP) system.

**Results:**

From August 2023 to July 2025, 15,837 requests for STI testing were made through GetaKit.ca, with testing requests increasing by 264% from year 1 to year 2. The overall positivity rate was 3.8%, remaining stable across both years. Total GetaKit costs were $248,098 in year 1 and $427,914 in year 2, corresponding to $72.73 and $34.44 per person tested, respectively. Comparable per-person costs were $49.39 under OHIP in both years and $68.59 (year 1) and $54.95 (year 2) in the STI clinic model. Although implementation-year costs were higher with the GetaKit model, per-person costs declined by more than 50% in year 2, resulting in savings of $14.95–$20.51 per test compared with traditional models.

**Conclusion:**

Together, these findings support online STI platforms as a cost-efficient complement to in-person services that can enhance timely diagnosis and treatment while helping to reduce longer-term healthcare costs associated with untreated infections.

## Introduction

In Canada, the rates of sexually transmitted infections (STIs) have increased,^1^ and health services are struggling to meet the demand for testing and treatment. While the COVID-19 pandemic led to a rise in virtual care,^2^ in which healthcare professionals (HCPs) provide services in real-time by phone or video, a newer alternative is asynchronous care through online platforms. Via asynchronous systems, individuals submit information through a website or digital application at one time, and, at a separate time, HCPs log into the system and review this information; real-time interaction with HCPs does not occur.^3^ One advantage of asynchronous systems is that individuals can request services at any time, and HCPs can review the submitted information during work hours.

One such model for asynchronous STI services is GetaKit, a study website through which individuals in Ontario, Canada can obtain testing, as clinically indicated from their local STI clinic.^4^ To use GetaKit, individuals go to the website (GetaKit.ca) and complete a risk assessment. Using clinician-developed computer algorithms and rule-based logic,^5^ the website processes these user submissions to offer each individual tailored testing options, and individuals opt in or out of each recommended test. Thereafter, nurses review these orders. Individuals then go to a lab for testing, and the labs report results back to both the GetaKit system (for individuals to view their results) and to the local STI clinic.^4^ Any individual with positive test results receives an appointment at their local STI clinic for same or next day services. GetaKit is thus asynchronous virtual access to testing – and treatment and follow-up when needed – from local bricks-and-mortar STI clinics.

We launched GetaKit as a research study in 2020 to evaluate a new access approach for HIV testing when in-person services were greatly reduced due to the COVID-19 pandemic.^6^ Building on early successes, we expanded GetaKit to include full lab-based STI testing. We have previously published on the GetaKit algorithms,^5^ diagnostic outcomes,^4,7^ and uptake of testing in general and in specific populations^4,8,9^; we have never reported a cost analysis. In this paper, we sought to determine the costs associated with operating GetaKit, compared to traditional clinical services. To complete this review, we present two years of STI testing data from GetaKit, detailing the costs to operate the system per patient tested. We then compare GetaKit costs that would have been incurred to provide STI testing to the same volume of patients in (1) our STI clinic and (2) via the publicly funded system in our jurisdiction. This public remuneration mechanism, known as the Ontario Health Insurance Plan (OHIP), covers full costs for STI testing and treatment services, including medication costs for STIs.

## Methods

At the time of publication, GetaKit is an ongoing prospective observational open-cohort study in Ontario, Canada. As the study is ongoing, open-ended, and observational, a sample calculation is not relevant.

We launched GetaKit in July 2020 to provide free HIV self-tests. In June 2023, we expanded the system to offer STI testing, including urine and oral/rectal swab testing for gonorrhea/chlamydia, blood testing for HIV, syphilis, and hepatitis C, and self-testing for HIV. To be eligible for STI testing through GetaKit, individuals had to be residing in Ontario, aged 17 years or older, and have risk factors for STI or HIV acquisition (whether through sexual contact or drug use practices). Testing recommendations in GetaKit align with guidelines from Public Health Ontario^10^ and the Public Health Agency of Canada,^11^ and people can retest at the frequencies listed in these guidelines.

### Clinical Flow

To obtain testing, an interested individual visits the website and registers, which involves providing a full name, contact details (email and phone number, both validated for two-factor authentication), and address. Once registered, people complete a one-time survey about sex and gender, sexual orientation, race and ethnicity, and country of birth. Each time individuals want STI testing, they complete a new STI risk assessment, which asks about sex and drug use practices, and STI testing histories and diagnoses. We created computer algorithms and rule-based logic that use individuals' information to provide individualized testing recommendations to them, per guidelines. Individuals then opt in or out of each recommended test and submit their requests. Nurses review each testing request to ensure it complies with guidelines. Approved requests are either digitally faxed to a local lab or mailed out. (Swabs are mailed out, whereas urine and blood-based testing only require requisitions, which can be digitally transferred to the lab.) Individuals then attend a local lab to drop off swabs and/or have blood and urine collected. The labs process these submitted samples and report results to GetaKit and to the local STI clinic. Nurses at GetaKit review these results and release them to individuals through the website. Nurses from the STI clinics contact individuals with positive test results to provide treatment, complete partner follow-up, and arrange retesting as needed.

### Data Collection

All data were collected through the website. Each order was tracked as a unique entry in the system’s database, and included data on risk factors, ordered tests, delivery method, and test results. These data were exportable into an anonymized CSV file.

### Data Analysis

We exported data for the period of August 1, 2023 to July 31, 2025. We first examined overall uptake over these 24 months and then separated this period, which allowed us to refine our cost analysis for our initial year of offering STI testing, compared to the second year when the system was better known. The data we extracted for analysis included: number of orders per period, method of delivery (digital versus mailout), and number of participants diagnosed with an STI.

To compare costs between GetaKit, the STI clinic, and the OHIP-funded services, we used the number of people tested through GetaKit as the sample size and used this figure to estimate (1) how much it cost overall for each 12-month period per model and (2) how much it cost to test a single patient through each model per 12-month period. Thus, we calculated the cost to test one individual through each model.

To determine costs for GetaKit, we used the yearly staffing costs and the per-item costs for shipping and specimen collection at the lab. For the STI clinic, we used the time allotment per appointment and the staffing costs associated with the number of hours of nursing time that would be required to see this volume of patients. The STI clinic does not have any associated lab fees. For the OHIP system, we used the cost for delivering STI testing to the volume of patients tested via GetaKit, plus the associated lab collection fees, as dictated by the OHIP fee schedule.^12^ For each model, we also added the associated costs for treating individuals who were diagnosed with an STI. For this, we took the number of STI diagnoses from the GetaKit data and added staffing costs. For the OHIP system and the STI clinic, we assumed that participants who had reported being an STI contact would have been treated empirically, following guidelines. For GetaKit, 100% of these patients required visits, as none had been seen in-person and therefore none would have received empiric treatment per guidelines. Lastly, we divided the total costs for GetaKit, the STI clinic, and the OHIP system by the number of patients who were tested to calculate a cost-per-patient tested. We completed these analyses for each 12-month period.

We also calculated positivity rates for GetaKit and did so by dividing the total number of unique infections that were identified by the total number of STI testing orders. As such, a person whose tests were positive for chlamydia in more than one anatomic site at a single testing time would only count as one infection, but this person may have been counted in the denominator more than once if they retested within the period of data analysis.

### Ethics and Funding

The Research Ethics Board at the University of Ottawa approved this project. Funding was obtained from the Ontario HIV Treatment Network, Public Health Ontario, and the Public Health Agency of Canada. Funders did not have any input on this publication.

### Conflict of Interest

The authors have no conflicts to declare.

## Results

From August 1, 2023 to July 31, 2025, there were 22,673 requests for testing via GetaKit, with 95.1% (n=21,553/22,673) of these requests being eligible. Overall, 69.8% (n=15,837/22,673) of these requests were for STI testing, with 30.2% (n=6794/22673) being for HIV self-tests only. When focusing on orders that included STI testing (including or excluding the HIV self-test), there were 3,411 requests in year 1 and 12,426 requests in year 2, representing a 264.3% increase from year 1 to year 2. Of all requests for STI testing, 35.3% (n=5,593/15,837) required mailout for swabs or an HIV self-test; the remainder required only that digital requisitions were provided to participants via their online account or faxed directly to the lab. Lastly, 597 participants had positive test results for an STI, for an overall positivity rate of 3.8% (n=597/15,837). The positivity rate was 3.9% (n=132/3411) in year 1 and 3.7% (n=465/12,426) in year 2. See Table 1 for a breakdown of the uptake data by year, showing total orders, total eligible orders (in that, there was a clinical indication for testing), total who sought only the HIVST and STI/HIV testing, and diagnosis numbers.

**Table 1.** GetaKit Uptake by Year

​	Year 1	Year 2
Total orders	8073	14600
Eligible	7563	13990
HIVST only	4125	2669
STI/HIV	3411	12426
Shipped (of STI/HIV)	1988	3605
Cases (all)	132	465
Cases (Not contacts)	98	358

We calculated the costs associated with the volume of testing that occurred through GetaKit per year (year 1 versus year 2) using (1) our expenditures to operate GetaKit, (2) the costs for nurses in our STI clinic to see that number of patients, and (3) the fees that would be incurred by the OHIP funded system to test that volume of patients. See Table 2.

**Table 2.** Calculated Costs

​	GAK		OHIP		SHC	
Costs	Year 1	Year 2	Year 1	Year 2	Year 1	Year 2
Staffing	$122,500	$185,000	$129,447	$471,567	$167,500	$612,500
Lab fees	$36,702	$133,704	$36,702	$133,704	$0	$0
Extras	$1,491	$2,704	$0	$0	$0	$0
Shipping	$12,425	$22,531	$0	$0	$0	$0
Treatment visits	$1,980	$6,975	$2,328	$8,503	$1,470	$5,370
Admin staff	$65,000	$65,000	$0	$0	$65,000	$65,000
Website operations	$8,000	$12,000	$0	$0	$0	$0
*TOTAL*	*$248,098*	*$427,914*	*$168,477*	*$613,773*	*$233,970*	*$682,870*
*Cost per person tested*	*$72.73*	*$34.44*	*$49.39*	*$49.39*	*$68.59*	*$54.95*

For GetaKit, costs arose from staffing, mailout fees (for envelopes, printed instruction sheets, shipping labels, and courier costs), website maintenance and hosting costs, lab fees charged to the OHIP system for testing, and the cost for nursing time at the STI clinic visits to treat patients who tested positive. For year 1, the staffing costs included 0.5 of a fulltime equivalent (FTE) for a nursing position (FTE = $125,000/year), totalling $62,500; 0.4 FTE of a nurse practitioner position (FTE = $150,000/year), totalling $60,000; and 1.0 FTE administrative staff ($65,000). Year 1 staffing costs were thus $182,500. In the second year, staffing costs were 1.0 FTE for a nursing position ($125,000); 0.4 FTE for a nurse practitioner position ($60,000); and 1.0 FTE for administrative staff ($65,000). Year 2 staffing costs totalled $225,000. In both years, mailout costs totalled $6.25 per package mailed, website fees were $8000 for year 1 and $12,000 for year 2, and lab costs were $10.76 per patient tested in both years. Because no patient was treated at an initial GetaKit visit, 100% of patients with an STI diagnosis needed to be seen for treatment. This added $1,980 in nursing costs for GetaKit in year 1 and $6,975 in year 2. Together, the total costs to the healthcare system were $248,098 in year 1 and $427,914 in year 2, resulting in a cost per-person-tested of $72.73 in year 1 and $34.44 in year 2.

Testing the same volume of patients through the publicly funded system as were tested via GetaKit would have incurred costs arising from the following fee codes: $37.95 per patient visit for testing, $10.76 per patient lab visit, and $23.75 per patient treatment visit for those diagnosed with an STI. Because these costs were stable, the cost per patient tested was $49.39 for both years. Guidelines recommend empiric treatment at the time of testing for patients who report being a sexual contact of someone diagnosed with an STI. Therefore, only patients who were not known contacts when they sought testing would have had to return to clinic for treatment. This additional treatment cost would have been $2328 in year 1 and $8503 in year 2. Thus, to test and treat the volume of patients seen via GetaKit through OHIP funding, the total costs to the healthcare system would have been $168,477 in year 1 and $613,773 in year 2.

For the STI clinic, costs arise from staffing. Currently, clinical visits with a nurse are scheduled for 30 minutes. We calculated STI clinic staffing costs by converting the number of patients into clinical time and then into fulltime equivalents (at $125,000 per FTE). For year 1, 3411 patients were seen. It would take 1705.5 nursing hours to accommodate this volume of patients in appointments. While one fulltime equivalent is 1820 hours per year, this number of hours is reduced by 4 weeks of vacation, 10 days of statutory holidays, and 10 days of sick time; this removes 336 hours, leaving 1484 hours per FTE. A total of 1705.5 nursing would thus require 1.15 FTEs nursing time, or $143,625 in salary. Additionally, it would take approximately 5 minutes per patient for the nurses to manage their lab results, once these return to the clinic. For year 1, this would add 284.25 hours of time. For year 2, the patient volume of 12,426 would require 6213 nursing hours, or 4.19 FTEs, costing $523,333. Added to this is the lab management time, of 1035.5 hours. For treatment costs, there would be an additional $1470 in year 1 and $5370 in year 2. For each year, an additional $65,000 for administrative staff would be required. The cost per patient tested would therefore have been $68.59 in year 1 and $54.95 in year 2. Year 1 total costs would have been $233,975 and year 2 total costs would have been $682,870.

## Discussion

In this paper, we reported on the first two years of offering STI testing via GetaKit, focusing on testing volume and associated costs. We then calculated the costs of offering the same volume of testing had it occurred (1) in our STI clinic and (2) through the local publicly funded (OHIP) system. Over this two-year period, there were 15,837 requests for STI testing via GetaKit, of which 78% (n=12,426) occurred in year 2. From this volume, we observed an overall positivity rate of 3.8% (n=597), with 132 diagnoses in year 1 and 465 in year 2. Despite a 264% increase in uptake in year 2, positivity rates remained relatively stable (3.9% in year 1; 3.7% in year 2). When we calculated the price per patient tested through each clinical modality, GetaKit cost $72.73 in year 1 and $34.44 in year 2; by comparison, it would have cost $49.39 in both years through OHIP, and $68.59 in year 1 and $54.95 in year 2 at the STI clinic. These findings raise several points for consideration.

First, our results suggest that asynchronous online systems can offer cost-effective STI screening but that these economic benefits have some caveats. Chief among these nuances is that systems, such as GetaKit, require a certain number of patients to access STI testing services to make them cost-effective. While the required patient volume to yield cost-savings will differ depending on the context of a given region (e.g., based on the cost of healthcare delivery and infection prevalence), simply hosting a website and having people register for services is likely not enough to achieve cost savings; a sufficient volume of patients must also seek testing to offset startup and operation costs.^13,14^ For GetaKit, we observed a more than 50% reduction in cost in the second year of operation compared to the first as a larger number of patients accessed the site for testing. Such successes required a certain level of (1) willingness on the part of individuals to access testing through an online system as opposed to in-person services, and (2) promotion by local partners of the system and its services to increase acceptance among potential users. Partnerships with public health units and STI clinics are an important part of our system, as these relationships provided local connections for treatment and other preventive services or referrals for those who required follow-up. We believe these partnerships in turn helped bolster uptake among individuals at risk for HIV and STIs.

Second, those implementing an online asynchronous system like GetaKit should expect that the system will be more costly during the implementation year. These increased costs may, as we observed, make the system substantially more expensive at the outset. As uptake and usage increases, however, these systems should become more cost-effective to the point that costs become lower in subsequent years due to the efficiencies inherent in this care delivery model. This point is reinforced by data from a 10-year retrospective economic evaluation^15^ of the Get Checked Online STI testing platform in British Columbia, Canada which noted cost-savings of $38.93 per testing episode for patients who accessed services online compared to an STI clinic and total provincial healthcare savings of over $1 million in 2023 (with these cost-savings increasing over time). In our case, after a single year, we observed cost savings of $14.95 per person tested. Economies of scale are thus key, and dedicated efforts are needed to reach the threshold at which costs fall below those incurred through traditional testing modalities.

Third, an important component in the cost-saving measures of online STI services is the use of automated algorithms for testing recommendations. As with GetaKit, we implemented a provider-developed process for STI screening^5^ based on individual risk practices and local guidelines and allowed individuals to select which tests (of those recommended) they wished to complete. Offering patients access to all available STI tests without clinical indications would likely result in unnecessary testing, incurring additional costs to the healthcare system^15^ (e.g., from performing clinically unnecessary tests) and to the online service itself (e.g., from added lab management or mailouts of swabs or other testing devices). Those seeking to implement online STI platforms should consider using screening algorithms to streamline testing and ensure that the process meets the needs of both individuals seeking, and HCPs providing, care.

Fourth, it is important to consider the advantages of asynchronous online STI testing systems to facilitate timely linkages to STI testing and the potential cost-savings that can arise from expedited diagnosis and treatment and the avoidance of sequelae from undiagnosed and untreated infections.^11^ In Ontario, Canada, for example, pelvic inflammatory disease secondary to untreated chlamydia or gonorrhea requiring hospitalization carries an estimated cost of $2.7 million annually to the provincial healthcare system ($4,800 estimated total cost per case multiplied by average volume of 569 cases).^16^ In addition, while diagnosis and treatment of uncomplicated infectious syphilis will incur specific costs, these costs are amplified in the context of untreated syphilis infections causing neurologic or ophthalmic involvement (neurosyphilis) or untreated infections during pregnancy leading to congenital infections in infants.^11^ Related to neurosyphilis management, previous research on the cost-effectiveness of syphilis screening among men who have sex with men in Ontario, Canada reported an estimated cost of $14,680 ($7,340-$22,020).^17^ This research, however, was completed in 2014 – and thus, costs in the current context are likely at the higher end of the estimated range. For congenital syphilis management, recent research conducted in Manitoba, Canada estimates the cost of treating one uncomplicated congenital syphilis case to be $18,151.40, including specialist consultation, intravenous treatment, and in-patient care fees.^18^ These costs will increase for more complicated presentations. Finally, at a national level, in Canada, the estimated lifetime cost of managing a new HIV diagnosis has increased by 11% from previous estimates to approximately $1.4 million per person.^19^ These costs were at least $1,623 higher for those diagnosed in later stages of HIV infection who required more clinical care, including hospitalization.^19^ Together, the costs of potential complications from untreated infections support the use of online STI testing systems to facilitate rapid linkages to testing and treatment, which in turn can help to reduce the cost burden to the healthcare system. While in-person care is recommended for patients with symptoms, asynchronous online systems can help to ensure appropriate clinical linkages without delaying care when clinical services are unavailable or inaccessible.

### Limitations

Our results must be interpreted in light of certain limitations. First, we did not include structural costs associated with the STI clinic, which GetaKit relies on to treat patients who test positive for an STI or HIV. Including these costs would have increased the total for each system. Second, we did not include the costs associated with providing healthcare to members of populations who typically face greater barriers to accessing care, including rural, Indigenous, people of colour, and gay, bisexual, and trans gender individuals. The costs associated with providing care to first-time testers were also not considered in this analysis. While these items would likely have affected our analyses, the trends are likely to remain unchanged: at a certain volume of usage, automated asynchronous systems to offer STI testing can be more cost-effective than testing through traditional healthcare and STI clinic settings. Third, our cost estimates are for uncomplicated testing and diagnostic episodes among adults (17 years of age or older) accessing services through public healthcare settings. Approximate cost burdens and savings would likely differ for clinical presentations or diagnoses in pregnant women and/or children and for those requiring specialist consultation, tertiary care visits, and/or in-person hospitalization. Fourth, our study does not directly address the care needs of people without Internet access or with disabilities that might preclude their abilities to use such online systems. However, we do not feel that online testing systems like GetaKit are well designed for the provision of such care. Instead, we suggest that online systems like ours can be used to divert individuals who are able to use them, so as to allow in-person care to be redirected toward individuals with greater equity disparities and barriers to care. In other words, we do not advocate for replacing in-person care with online services, but rather, to use this to supplement in-person services so as to re-orient in-person care to the individuals who would benefit most from such access.

## Conclusions

Given rising STI rates and limited access to clinical screening services, particularly in the absence of symptoms, we developed an asynchronous platform (GetaKit.ca) that allows individuals to obtain STI screening without visiting a clinic. In this analysis comparing healthcare costs between GetaKit and in-person clinical services, we found our online platform generated savings of $14.95 to $20.51 after at least two years of operation and a more than 50% reduction in costs between the first and second years of implementation, and we anticipate these costs per person tested will continue to decline over time. In addition to overall cost savings, this platform also enabled rapid access to local testing, diagnosis, and treatment, thus reducing the potential for sequelae from untreated infections.

## Data Availability

Upon reasonable request.*
